# Energy-efficient Area Coverage by Sensors with Adjustable Ranges

**DOI:** 10.3390/s90402446

**Published:** 2009-04-08

**Authors:** Vyacheslav Zalyubovskiy, Adil Erzin, Sergey Astrakov, Hyunseung Choo

**Affiliations:** 1 Sobolev Institute of Mathematics, Siberian Branch of the Russian Academy of Sciences, Novosibirsk, Russia; E-Mails: slava@math.nsc.ru (V.Z.); adilerzin@math.nsc.ru (A.E.); 2 Russian State University of Trade and Economics, Kemerovo Branch, Kemerovo, Russia; E-Mail: astrakov90@gmail.com (S.A.); 3 School of Information and Communication Engineering, Sungkyunkwan University, Suwon, Korea

**Keywords:** Coverage, deployment, energy efficiency, wireless sensor networks, simulation

## Abstract

In wireless sensor networks, density control is an important technique for prolonging a network’s lifetime. To reduce the overall energy consumption, it is desirable to minimize the overlapping sensing area of the sensor nodes. In this paper, we study the problem of energy-efficient area coverage by the regular placement of sensors with adjustable sensing and communication ranges. We suggest a more accurate method to estimate efficiency than those currently used for coverage by sensors with adjustable ranges, and propose new density control models that considerably improve coverage using sensors with two sensing ranges. Calculations and extensive simulation show that the new models outperform existing ones in terms of various performance metrics.

## 1. Introduction

A wireless sensor network (WSN) is composed of a large number of sensor nodes that are densely deployed near an area of interest and are connected by a wireless interface. Since each sensor is equipped with a limited power source and, in most applications, it is impossible to replenish power resources, a major constraint of WSN lifetime is energy consumption. Energy savings optimization is thus a major challenge for the success of WSNs. Typical tasks of a sensor node in a sensor network are to collect data, perform data aggregation, and then transmit data. Among these tasks, monitoring and transmitting data require much more energy than processing it [1]. Generally, there are two basic approaches to the problem of saving energy in WSN. The first one is scheduling some sensor nodes to go into an active mode while enabling the other sensor nodes to go into a low-power sleep mode [2,3]. The second approach is to adjust the transmission or sensing ranges of the sensor nodes, eliminating redundant energy consumption [4,5].

Coverage is one of the most important issues of the WSN. Since sensors have limited battery life, wireless sensor networks are characterized by high node density. It is not necessary to have all sensor nodes operate simultaneously in active mode and different scheduling methods are used to ensure energy-efficient coverage and connectivity [6]. The problem we study is how to arrange the sensors in the nodes of a regular plane grid and how to set the sensing range of each sensor to minimize the redundantly covered area. It was shown in [7] that if all the sensors have the same sensing range and communication range, then each of the three closest nodes should form an equilateral triangle to minimize the overlap of the sensing areas. In [8], the result was generalized by considering sensors with various sensing ranges, and two new density control models were proposed.

In this paper, we suggest a more accurate method for estimating energy efficiency based on coverage of the sensing area by equal, non-overlapping standard figures (*tiles*), and we propose two types of density control models. Similar to [8], we suppose that each sensor with a certain sensing range determines a disk centered at the sensor, and we consider the two types of coverage models. In the first-type models, the centers of the three neighboring disks with the same radius are placed at the vertices of an equilateral triangle, and form a uniform triangular grid. In the second-type models, the centers of four neighboring disks with the same radius are at the vertices of a square, and form a homogeneous rectilinear grid. We formulate and solve a special optimization problem that allows us to propose new models in which the sensing energy consumption is reduced substantially with a high degree of coverage provided.

The rest of the paper is organized as follows. Section 2 presents related work. In Section 3, we introduce several coverage models and give theoretical estimates of their energy efficiency. Section 4 contains the simulation and performance evaluation results. Section 5 concludes the paper.

## 2. Related Work

The coverage problem is a key issue for any wireless sensor network, and coverage can be viewed as one measurement of quality of service of the system. Most sensor networks have both high node density and limited node power. The goal is to minimize energy consumption to prolong the system’s lifetime, while maintaining effective coverage. Coverage can be achieved by designing some kind of density control mechanism, that is, scheduling the sensors to work alternatively to minimize the power wastage due to the overlap of active nodes’ sensing areas.

Many topology and density control methods have been proposed for wireless sensor networks. In [9] the problem of finding the maximal number of covers in a sensor network was considered. Here a cover is a set of nodes that can completely cover the monitored area. It was proved that the problem is NP-complete and several methods were developed to solve this problem approximately. In [10], GAF self-configures redundant nodes into small groups based on their location and uses localized algorithms to control node duty cycle to extend network lifetime. This method can guarantee connectivity, but not complete coverage. In PEAS [11], sleeping nodes wake up once in a while to broadcast a probing message over a certain range and to replace any failed working node. The probing range can be adjusted to achieve different levels of coverage overlap, but it also cannot ensure complete coverage. To provide complete coverage, in [12] a sponsored area algorithm is developed that uses the following off-duty eligibility rule. A node can turn itself off and sleep as long as its working neighbors can cover all of its sensing area. This rule underestimates the area already covered, leading to excessive energy consumption.

It is proven that if the radio range is at least twice the sensing range, complete coverage of a convex area implies connectivity among the working set of nodes [7]. Thus, when the condition is satisfied, the topology control problem is simplified and becomes a sensing coverage problem. It is well known that placing disks on the vertices of a regular triangular lattice is optimal in terms of the number of disks needed to achieve full coverage of a plane. Its asymptotic optimality was proved in [13], and was recently proven again in [7] using a different approach. Based on these results, the authors of [7] introduced a distributed density control algorithm named OGDC. In the case when all the nodes have the same sensing range *r_s_*, every triplet of closest nodes in a cover can form an equilateral triangle with the side length $\sqrt{3}r_{s}$. In this way the overlap of sensing areas of all the nodes is minimized. Simulation results show that OGDC exhibits better performance than other algorithms with respect both to coverage and energy consumption.

Most density control algorithms assume that the sensing ranges of all sensors are the same. In [7], the authors extend the original node-scheduling model to include different sensing ranges. The problem they try to deal with is how to let the model work when different sensor nodes have different sensing ranges, but not to exploit the adjustable sensing ranges to achieve better performance. The authors of [8] utilized sensing range adjustability to design the node-scheduling scheme to minimize the energy consumption as much as possible and achieve a longer living sensor network. As opposed to the uniform sensing range model of [7], they proposed two other node scheduling models with several levels of adjustable sensing ranges.

## 3. Proposed Coverage Models

We assume the sensor nodes are randomly deployed over a two-dimensional square area, and that the location of each node is known. The sensing area of a node is a disk of a given radius (sensing range). To guarantee network connectivity, we assume that all the active sensor nodes form a minimal spanning tree, and that each sensor node adjusts its communication range to reach its furthest neighbor on the tree.

### 3.1. Coverage with Uniform Sensing Range

**Figure 1.**
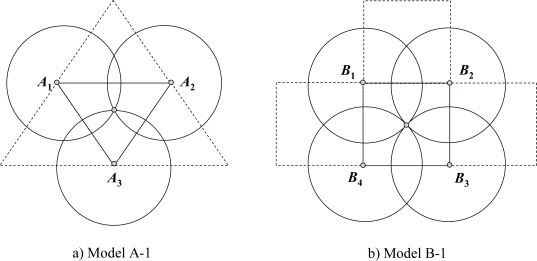
Coverage with uniform sensing range.

Suppose first that the sensing ranges of all sensors are equal, and that it is necessary to cover every point of the monitoring area by at least one disk of radius *R*. In model A-1, any three neighboring disks have exactly one common point, and by connecting the centers of neighboring disks, one gets a uniform triangular grid (see Figure 1a). In model B-1, there is exactly one common point for every four neighboring disks of radius *R*, and if one connects the neighbor center nodes of disks, then one gets a uniform rectilinear grid (see Figure 1b).

To estimate the efficiency of the coverage models, we use regular polygons - tiles that cover the whole monitoring area without overlapping. For model A, the tile is a triangle *A_1_A_2_A_3_*, and for model B, the tile is a square *B_1_B_2_B_3_B_4_* (see Figure 1). Since in the real case the monitoring area is sufficiently larger than the sensor’s sensing disk, we can ignore an edge effect during the estimation of coverage efficiency and suppose that all tiles are covered in the same manner. Then for a given cover, we define the *coverage density D*, the ratio of the total area *Sf* of the parts of disks inside the tile divided by the area *Sp* of the tile. Obviously the lowest possible value of *D* is one. The smaller value of *D* corresponds to the better (energy-efficient) cover. For model A-1 the corresponding areas and the coverage density are:

$${\mathrm{Sf}}_{A-1}=\pi R^{2}/2,\;\;\;{\mathrm{Sp}}_{A-1}=3R^{2}\;\sqrt{3}/4,$$

$$D_{A-1}={\mathrm{Sf}}_{A-1}/{\mathrm{Sp}}_{A-1}=2\pi /(3\sqrt{3})\approx 1.2091.$$

For model B-1 the areas and the coverage density are:

$${\mathrm{Sf}}_{B-1}=\pi R^{2},\;\;\;{\mathrm{Sp}}_{B-1}=2R^{2},$$

$$D_{B-1}={\mathrm{Sf}}_{B-1}/{\mathrm{Sp}}_{B-1}=\pi /2\approx 1.5708.$$

Similar to [8], we suppose that the sensing energy consumption is proportional to the area of sensing disks by a factor of *μ*_1_, or the power consumption per unit. Then, the *sensing energy consumption per (unit) area* (SECPA) is *E* = *μ*_1_ · *D*, and for model A-1 and model B-1 we have:

$$E_{A-1}=\mu_{1}2\pi /(3\sqrt{3})\approx 1.2091\mu_{1},\;\;\;E_{B-1}=\mu_{1}\pi /2\approx 1.5708\mu_{1}.$$

A similar characteristic is calculated in [8] for model A-1, where it is called Model-I, as follows:

$$E_{I}=\mu_{1}6\pi /(4\pi +3\sqrt{3})\approx 1.0615\mu_{1}.$$

This is apparently incorrect, because not all overlap of the disks was considered. In model A-1, each disk intersects with six other disks, but in [8] the authors consider only two overlaps. In our case, we consider all overlaps inside the tile, and since the tiles cover the whole monitoring area without overlapping, our calculation is more accurate. Note that the minimal number *N* of sensor nodes needed to cover some area is given by the equation:

$${\mathrm{NR}}^{2}\pi /S=2\pi /\sqrt{27}\approx 1.2091,$$

where *S* is the size of the monitored area and *R* is the sensing range [14]. Then, the minimal SECPA in model A-1 is in line with the results in [7].

Model B-1 was not considered in [8], but as will be demonstrated, a square grid-based model exhibits a good coverage density after some modifications. Moreover, a rectangular placement grid seems to be more convenient in practice, especially in the case of covering a rectangle area.

### 3.2. Coverage with Two Adjustable Sensing Ranges – Tangent Disks

**Figure 2.**
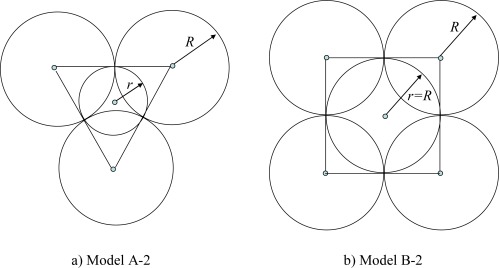
Coverage with two sensing ranges.

If every three equal neighboring disks in model A (or four equal neighboring disks in model B) are tangent, then there is a gap between them (see Figure 2). That uncovered space may be covered by one *extra disk*. When doing so, we refer the models as models A-2 and B-2, respectively.

It is easy to check that for model A-2, the radius of the extra disk is $r=R/\sqrt{3}$, and for model B-2 the radius of extra disk is *r* = *R*. The coverage density of model B-2 is the same as for B-1, but that of A-2 differs from that of A-1:

$${\mathrm{Sf}}_{A-2}=5\pi R^{2}/6,\;\;\;{\mathrm{Sp}}_{A-2}=R^{2}\sqrt{3},$$

$$D_{A-2}={\mathrm{Sf}}_{A-2}/{\mathrm{Sp}}_{A-2}=5\pi /(6\sqrt{3})\approx 1.5115,$$

$$E_{A-2}=\frac{\mu_{1}5\pi }{6\sqrt{3}}\approx 1.5115\mu_{1},\;\;\;E_{\mathrm{II}}=\frac{\mu_{1}\pi (3R^{2}+r^{2})}{(\sqrt{3}+5\pi /2)R^{2}}\approx 1.0924\mu_{1}.$$

The coverage density in model A-2 is substantially increased compared to model A-1. Model A-2 was also considered in [8] (there it was called Model-II), and the difference between *E_A-2_* and *E_II_* is even larger than that between *E_A-1_* and *E_I_*. The calculation of *E_II_* in [8] seems to be incorrect for the same reason as for Model-I.

### 3.3. Optimal Arrangement of Sensors with Two Adjustable Sensing Ranges

If the energy consumption of a disk of radius *r* is proportional to *r^n^*, *n* ≥ 2, then one could save energy by decreasing the radius of an extra disk. In order to decrease the radius *r* of an extra disk and to preserve the coverage, the neighboring disks of radius *R* must overlap. Though this overlapping may be small, the radius *r* may decrease gradually. We seek an *optimal* radius *r* for the extra disk, and this determines the extent to which the disks of radius *R* overlap. This case is intermediate between the previous models, and we will illustrate its advantage over them.

**Figure 3.**
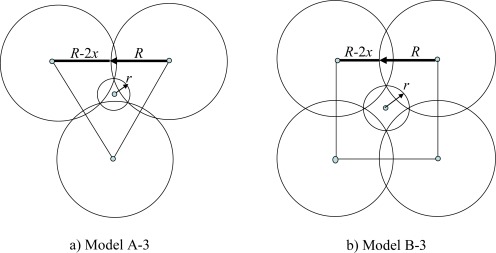
Optimal coverage with two sensing ranges.

In model A-3 we permit equal overlapping of the neighboring disks of radius *R* and seek the radius *r* of an extra disk which minimizes the SECPA. The tile for model A-3 is an equilateral triangle with vertices in the three neighboring disks of radius *R* (see Figure 3a). The problem is to determine the placement of the disks of radius *R* which minimizes energy consumption. Note that the radius of an extra disk *r* is defined explicitly by the placement of the bigger disks.

**Theorem 1:** The optimal placement of disks in model A-3 is determined by the following values:

$$r=R/\sqrt{31}\approx 0.1796R;\;\;\;\;a=6R\sqrt{3}/\sqrt{31}\approx 1.8665R,$$

where *a* is the distance between the centers of neighboring disks of radius *R* (equivalently, the side length of the triangular tile).

**Proof:** Let us denote *x* = *R* − *a*/2. Then $r=t/\sqrt{3}-\sqrt{R^{2}-t^{2}}$, where *t* = *R* − *x*. And we have

$$\mathrm{Sp}(t)=a^{2}\;\sqrt{3}/4={\left(R-x\right)}^{2}\;\sqrt{3}=t^{2}\;\sqrt{3},\;\;\;\;\mathrm{Sf}(t)=\pi R^{2}/2+\pi {\left(t/\sqrt{3}-\sqrt{R^{2}-t^{2}}\right)}^{2}.$$

In order to minimize the sensing energy consumption per unit area, it is necessary to solve the following optimization problem:

$$D(t)=\frac{\mathrm{Sf}\;(t)}{\mathrm{Sp}\;(t)}=\frac{\pi }{\sqrt{3}}\left(\frac{3R^{2}}{2t^{2}}-\frac{2}{3}-\frac{2\sqrt{R^{2}-t^{2}}}{t\sqrt{3}}\right)\to \underset{t}{\text{min}}.$$

The solution of this problem is $t=3R\sqrt{3}/\sqrt{31}$, and the other values are:

$$x=R-t=R(\sqrt{31}-3\sqrt{3})/\sqrt{31},\;\;\;r=t/\sqrt{3}-\sqrt{R^{2}-t^{2}}=R/\sqrt{31},\;\;a=6R\sqrt{3}/\sqrt{31}.$$

As a result, we have ${\mathrm{Sp}}_{A-3}=27R^{2}\sqrt{3}/31$, *Sf*_*A*−3_ = 33*πR*^2^/62, $D_{A-3}=11\pi /(18\sqrt{3})\approx 1.1084$, and *E_A-3_* ≈ 1.1084*μ*_1_. The radius *r* of the extra disk in model A-3 is approximately 17 percent of radius *R*, yielding a substantial reduction of sensing energy consumption per unit area. Since energy consumption is proportional to *r^n^*, *n* ≥ 2, this gain increases with *n*.

In model B-3, the tile is a square with vertices in the centers of four neighboring disks of radius *R* (see Figure 3b). Note that in that case, the diagonal disks do not intersect, but all these four disks are the neighbors of an extra disk of radius *r*.

**Theorem 2:** The placement of disks in model B-3 minimizing SECPA is defined by the following values:

$$r=R/\sqrt{5}\approx 0.4472R,\;\;a=4R/\sqrt{5}\approx 1.7889R,$$

where *a* is the side of the square tile.

**Proof:** Let us denote *x* = *R* − *a*/2. Then $r=t-\sqrt{R^{2}-t^{2}}$, where *t* = *R* − *x*. Calculate

$$\mathrm{Sp}(t)=4{\left(R-x\right)}^{2}=4t^{2}\;\;\text{and}\;\;\mathrm{Sf}(t)=\pi R^{2}+\pi {\left(t-\sqrt{R^{2}-t^{2}}\right)}^{2}.$$

In order to minimize the SECPA, we need to solve the following problem:

$$D(t)=\frac{\mathrm{Sf}\;(t)}{\mathrm{Sp}\;(t)}=\frac{\pi }{2}\;\left(\frac{R^{2}}{t^{2}}-\frac{\sqrt{R^{2}-t^{2}}}{t}\right)\to \underset{t}{\text{min}}.$$

Its solution is $t=2R\sqrt{5}$, then $x=R-t=R(\sqrt{5}-2)/\sqrt{5}$, $r=t-\sqrt{R^{2}-t^{2}}=R/\sqrt{5}$, $a=2(R-x)=4R/\sqrt{5}$, and the theorem is proved.

As a result, we have *Sp*_*B*−3_ = 16*R*^2^/5, *Sf*_*B*−3_ = 6*πR*^2^/5, *D*_*B*−3_ = (3*π*)/8 ≈ 1.1788, and *E_B-3_* ≈ 1.1781*μ*_1_.

### 3.4. Coverage with Three Adjustable Sensing Ranges

**Figure 4.**
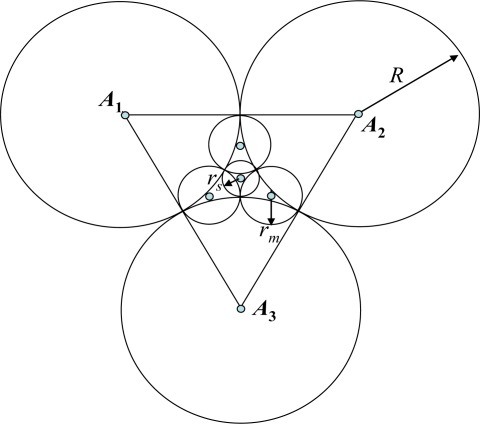
Model III [8].

Further improvement of the models is possible if one uses more sensing ranges, but sometimes this presents a negative effect. For example, in [8] the authors introduced Model III with three sensing ranges (see Figure 4), but the SECPA in Model III is even worse than in model A-1. The proper calculation of the coverage density for Model III is given below. The area of the triangular tile is ${\mathrm{Sp}}_{\mathrm{III}}=R^{2}\sqrt{3}$. The total area of three parts of disks of radius *R* inside the triangle is *πR*^2^/2. Taking into account that $r_{m}=(2-\sqrt{3})R$ and $r_{s}=(2/\sqrt{3}-1)R$, we conclude that the total area of covers inside the triangle is:

$${\mathrm{Sf}}_{\mathrm{III}}=\pi (R^{2}/2+3r_{m}^{2}+r_{s}^{2})\approx 0.7394\pi R^{2},$$

and the corresponding coverage density and SECPA are:

$$D_{\mathrm{III}}=\frac{{\mathrm{Sf}}_{\mathrm{III}}}{{\mathrm{Sp}}_{\mathrm{III}}}=\frac{\pi (R^{2}/2+3r_{m}^{2}+r_{s}^{2})}{R^{2}\sqrt{3}}\approx 0.7394\pi /\sqrt{3}\approx 1.3412,\;E_{\mathrm{III}}\approx \mu_{1}\cdot D_{\mathrm{III}}\approx 1.3412\mu_{1},$$

which is greater than *E*_*A*−1_ ≈ 1.2091μ_1_.

### 3.5. Communication Energy Consumption

Communication energy consumption depends on the distance between the communicating sensors. In order to estimate the *communication energy consumption per (unit) area* (CECPA), we assume that all sensors are involved in communication. Similar to [8], in order to find a communication graph, we construct a minimal spanning tree (MST) that spans all the sensors. We assume that the energy consumed by communication for a sensor is proportional to the square of the distance from itself to its farthest on the tree neighbor by a factor of *μ*_2_. Then, CECPA is the part of the sensors’ communication energy used by the nodes inside a tile divided by the tile’s area.

As for the estimation of coverage density, we ignore the edge effect and calculate CECPA for the case of infinite grid. Since in model A-1 the communication energy is used by the nodes of tile triangles, and the total number of tiles in the infinite triangular grid is twice the number of centers of disks of radius *R*, CECPA is half of the communication energy of one node divided by the tile’s area. The distance between any two adjacent nodes in MST in model A-1 is $d_{A-1}=R\sqrt{3}$. So, the CECPA in model A-1 is:

$${\mathrm{CE}}_{A-1}=0.5\mu_{2}\;d_{A-1}^{2}/S_{P_{A-1}}=2\mu_{2}/\sqrt{3}\approx 1.1547\;\mu_{2},$$

where the parameter *μ*_2_ is independent of distance. In model B-1, the communication energy is used by the nodes of the tile square, and the total number of square vertices in the infinite square grid is equal to the number of squares, and so in that case CECPA is equal to the communication energy of one node divided by the tile’s area. The distance between any two adjacent nodes in MST in model B-1 is $d_{B-1}=R\sqrt{2}$. Then CECPA in model B-1 is:

$${\mathrm{CE}}_{B-1}=\mu_{2}\;d_{B-1}^{2}/S_{P_{B-1}}=\mu_{2}.$$

In model A-2, during the construction of MST, every vertex of a tile triangle will choose the center of an extra disk to connect, and the center of that extra disk can choose either the center of a larger disk or the center of a smaller one. All these edges have the same length: $d_{A-2}=2R/\sqrt{3}$. Therefore, the CECPA is the communication energy of the extra sensor and half of the communication energy of one vertex of the tile triangle divided by the tile’s area, as shown below:

$${\mathrm{CE}}_{A-2}=(1+0.5)\;\mu_{2}\;d_{A-2}^{2}/S_{P_{A-2}}=2\mu_{2}/\sqrt{3}\approx 1.1547\;\mu_{2}.$$

In model B-2 all the edges in MST have the identical length $d_{B-2}=R\sqrt{2}$, so:

$${\mathrm{CE}}_{B-2}=(1+1)\;\mu_{2}\;d_{B-2}^{2}/S_{P_{B-2}}=\mu_{2}.$$

In model A-3 every vertex of a tile triangle will choose the center of an extra disk to connect, and the center of the extra disk will choose either the center of a larger disk or the center of a smaller one. Since all these edges have the identical length $d_{A-3}=6R/\sqrt{31}$, then:

$${\mathrm{CE}}_{A-3}=(1+0.5)\;\mu_{2}\;d_{A-3}^{2}/S_{P_{A-3}}=2\mu_{2}/\sqrt{3}\approx 1.1547\;\mu_{2}.$$

Finally, in model B-3, every vertex of a square tile will choose the center of an extra disk to connect, and the center of the extra disk will choose the center of a larger disk. The length of each edge is $d_{B-3}=2\sqrt{2}R/\sqrt{5}$, and then:

$${\mathrm{CE}}_{B-3}=(1+1)\;\mu_{2}\;d_{B-3}^{2}/S_{P_{B-3}}=\mu_{2}.$$

We have summarized the results for the considered models in Table 1.

As one can see, model A-3 is the best with respect to the sensing energy consumption per unit area, while the B series of models are the best ones with respect to the communication energy consumption per unit area. Since sensing is a permanent duty and communication occurs occasionally, we can conclude that models A-3 and B-3 are the best among the considered models.

## 4. Performance Evaluation

### 4.1. Parameters and Performance Metrics

For evaluation of our proposed models, we customize a simulator similar to that in [8]. Sensor nodes are randomly deployed in a 50 × 50 m^2^ area. In order to ignore the edge effect, we use the middle (50 − *R*) × (50 − *R*) m^2^ of the area to calculate the coverage ratio. We assume the sensing energy consumption of each sensor is proportional to the square of its sensing range. To estimate transmission energy, we first construct a minimal spanning tree among the working nodes. We assume that the energy consumed by communication for a working sensor is proportional to the *n*’s power of the distance to its farthest neighbor in the tree (*n* = 2, 4). Total energy consumption is calculated as a weighted sum of the sensing and communication energies. To denote the ratio of sensing energy to total energy consumption, we use a parameter *k*, 0 ≤ *k* ≤ 1.

For randomly deployed sensors we cannot guarantee that we will find a sensor at any desirable position, so in the simulation we choose the sensor node *closest* to the ideal position. The number of deployed nodes, *N*, varies from 200 to 1000. The sensing range of the large disk (with radius *R*), varies from 4 m to 12 m. A more detailed description of the simulation environment can be found in [8].

We use the same performance metrics as in [8]: (1) ratio of the covered area to the total monitored area (coverage ratio), (2) sensing energy consumption in one round, (3) communication energy consumption in one round, (4) and weighted sum of sensing and communication energy consumption as total energy consumption.

### 4.2. Simulation Results

**Figure 5.**
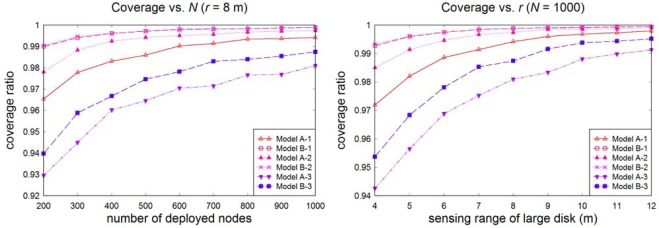
Coverage variations with different node density and sensing range.

Figure 5 shows the dependence of the coverage ratio on node density and sensing range. We can see from this that the coverage ratio is strongly correlated with SECPA: a smaller SECPA corresponds to smaller coverage ratio. The relatively energy-inefficient models B-1 and B-2 achieve a better coverage ratio, especially for low node densities and short sensing ranges. When node density is high or the sensing range is large enough, however, all the models will have good coverage performance.

**Figure 6.**
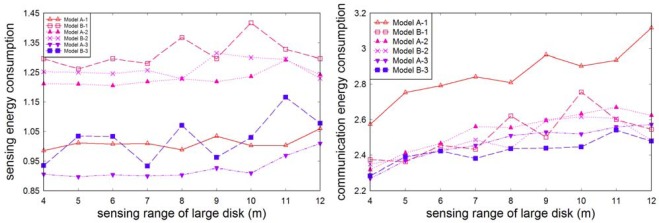
Energy variations with different sensing range (*N* = 1,000).

Figure 6 illustrates the energy consumption in one round for various sensing ranges. We can see that models B-1, B-2, and A-2 have greater sensing energy consumption than model A-1. The sensing energy consumption of model B-3 is very close to that of model A-1, and model A-3 outperforms all another models. Models A-3 and B-3 provide the best results in terms of communication energy consumption per unit area. The results fully agree with our theoretical estimates in Section 3.

**Figure 7.**
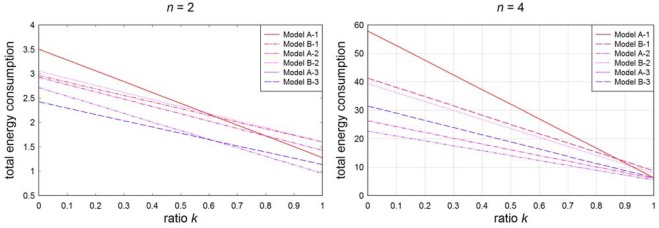
Total energy consumption with various ratios *k* (*r* = 8 m, *N* = 1,000).

Total energy consumption is shown in Figure 7. For the path loss exponent *n* = 2, models A-3 and B-3 perform best. In the case *n* = 4, A-3, B-3 and A-2 do best.

**Figure 8.**
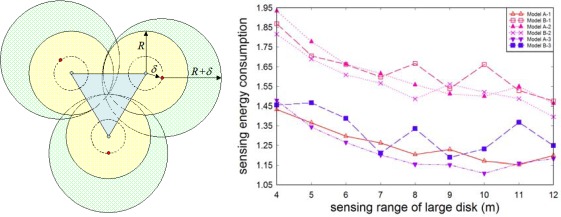
Modified model and sensing energy consumption with 100% coverage (*N* = 1,000).

To provide 100% coverage in the case of randomly deployed sensors, we modify all the models as follows. Each selected sensor node stretches its sensing range to its distance *δ* from an ideal position. Figure 8 shows the energy consumption of the modified algorithm for various sensing ranges. We can see that the ranking of the considered models in this case is very similar to that of the base model: sensing energy consumption of model B-3 is close to that of model A-1, and model A-3 provides the best result. Taking into account better communication energy consumptions in models A-3 and B-3, as compared to model A-1, we can conclude that models A-3 and B-3 remain more energy-efficient for this modification.

From this simulation we conclude the following:

- The coverage ratio of all considered models is proportional to the corresponding sensor energy consumption per unit area.
- Whereas models A-3 and B-3 exhibit slightly worse performances with respect to the coverage ratio, they provide necessary gains in terms of energy consumption. The modified version of the algorithm provided 100% coverage, and it also demonstrates the superiority of these two models. For example, for simulation parameters chosen based on the hardware of Crossbow MicaZ nodes the models show that energy consumption is reduced up to 28% for an indoor scenario (communication range is 8 m) and up to 19% for an outdoor scenario (communication range is 20 m).
- A larger path lost exponent yields greater energy savings in the proposed models.

## 5. Conclusions and Future Work

In this paper, we considered two types of sensor covers: model A and model B. In model A, the centers of three neighboring disks of equal radius are placed at the vertices of an equilateral triangle. In model B, the centers of four neighboring disks of equal radius are at the vertices of a square. For each type of cover, we considered three models: A-1, A-2, and A-3, and B-1, B-2, and B-3. Newly introduced models A-3 and B-3 bring about a significant improvement in coverage efficiency. We have proposed an accurate calculation of sensing and communication energy consumption per unit area by disks of two different radii. We formulated a correction for performance evaluation of models A-1 and A-2 from [8] as well. In terms of sensing energy efficiency, model B-3 is slightly less effective than model A-3, but it has several advantages due to its simpler grid structure. The location of the sensors is easier to find, and such coverage is more convenient for rectangle area coverage. Moreover, model B-3 has a smaller communication energy consumption per area. Extensive simulations prove that the two models also outperform the others in terms of energy consumption for randomly deployed nodes.

**Figure 9:**
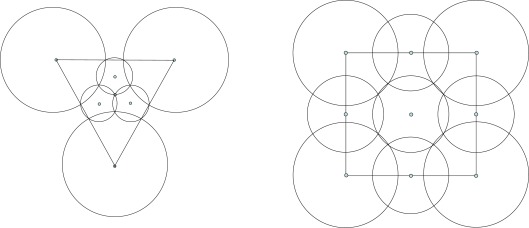
Separation of big disks.

Since the qualitative leap in models A-3 and B-3 was obtained by optimal overlapping of neighboring disks of radius *R*, for the sake of completeness one can vary the separation of disks of radius *R* and fill the empty space between them with disks of radius *r* (see Figure 9). We omit here the details and only indicate that such models are worse than models A-3 and B-3 with respect to SECPA in the case of two sensing ranges.

**Figure 10:**
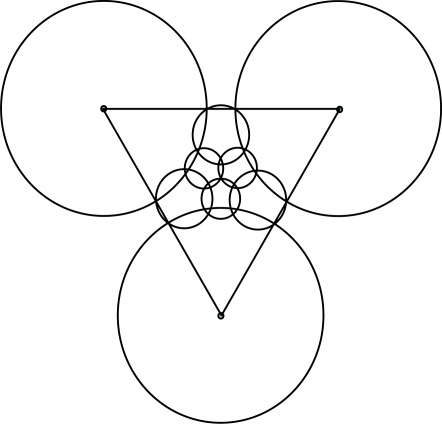
Coverage with three sensing ranges.

In the case of three or more sensing ranges, one can get noteworthy results by separating the disks of radius *R* (see Figure 10). In future research, we are planning to investigate coverage by sensors with three or more sensing and communication ranges.

In the paper, we assumed that all nodes in a sensor network have circular sensing regions. However, this assumption may not be accurate in real world networks. The considered models can be extended for the case when the shape of the covered region is an ellipsoid instead of a circle. If the ratio between corresponding semimajor and semiminor axes of the ellipsoids is the same for different power levels, and it is possible to give the same orientation to all sensors, we can extend our models using simple affine transformation. Optimality condition (the ratio between big and small radii) for models A-3 and B-3 remains the same. In case where the sensors cannot be oriented properly or nodes may have irregular sensing regions, the analysis of the presented models needs to be reexamined based, for example, on the values of minimum and maximum sensing ranges of a node [15]. In the future, we will extend our solution to handle more sophisticated sensing and communication models.

Our paper focuses on area coverage in random deployed WSN, where the density of static sensor nodes compensates for the lack of exact positioning. The primary goal of the paper is to estimate a potential of adjustable sensing ranges in terms of energy efficiency. In the future, we will consider other applications (target coverage, mobile sensors or targets). Finally, we want to set up experimental testbeds for further validation of our results.

## Figures and Tables

**Table 1:** Energy consumption per area for different models.

	**A-1**	**A-2**	**A-3**	**B-1**	**B-2**	**B-3**
SECPA	1.20*μ*_1_	1.51*μ*_1_	1.10*μ*_1_	1.57*μ*_1_	1.57*μ*_1_	1.17*μ*_1_
CECPA	1.15*μ*_2_	1.15*μ*_2_	1.15*μ*_2_	*μ*_2_	*μ*_2_	*μ*_2_
